# Beyond Survival: A Single-Center Analysis of Complication Burden, Healthcare Utilization, and Treatment Feasibility in Higher-Risk Myelodysplastic Neoplasms

**DOI:** 10.7759/cureus.106216

**Published:** 2026-03-31

**Authors:** Mihai-Emilian Lapadat, Oana Stanca, Irina N Triantafyllidis, Anca M Ciobanu, Nicoleta M Berbec, Cristina Negotei, Cristian T Barta, Andrei Colita

**Affiliations:** 1 Department of Hematology, Carol Davila University of Medicine and Pharmacy, Bucharest, ROU; 2 Clinic of Hematology, Coltea Clinical Hospital, Bucharest, ROU

**Keywords:** complication burden, healthcare utilization, higher-risk mds, hypomethylating agents, major bleeding, severe infection, transfusion burden, treatment feasibility

## Abstract

Background

Higher-risk myelodysplastic neoplasms (MDS) are commonly evaluated using overall survival (OS) and leukemic transformation, but these endpoints may underrepresent the day-to-day burden of cytopenia and treatment-related complications. We aimed to characterize complications as central outcomes in a real-world, single-center cohort of the Revised International Prognostic Scoring System (IPSS-R) high/very high-risk MDS and to quantify complications using a simple burden framework.

Methods

Retrospective study of adults with IPSS-R high/very high-risk MDS managed in a tertiary center. Infectious complications (any infection, severe infection, or sepsis); hemorrhagic complications (any bleeding or major bleeding); ICU admission; hospitalizations; hospital days; transfusion requirements; and cause of death were collected. A severe complication composite was defined as severe infection and/or sepsis, major bleeding, and/or ICU admission. A severe complication count captured the number of severe domains (zero, one, two-three). Healthcare utilization and transfusion burden were expressed as rates per patient-month. OS was assessed using Kaplan-Meier methods with log-rank testing; survivor-conditioned landmark-proxy analyses were performed at three and six months.

Results

Seventy-two patients were included (median age 71 years; 55.6% male); 45.8% were IPSS-R high and 54.2% very high. Hypomethylating agents (HMAs) were administered to 61.1%, and the median follow-up was 11 months. Complications were frequent: any infection occurred in 58.3%, severe infection in 36.1%, sepsis in 25.0%, any bleeding in 55.6%, major bleeding in 26.4%, and ICU admission in 16.7%. The severe complication composite occurred in 52.8%; the severe count was zero in 47.2%, one in 33.3%, and two-three in 19.4%. The severe composite was associated with inferior OS (median 11 vs. 16 months; p=0.0238), and OS worsened across severe-count categories (16 vs. 12 vs. nine months; p=0.0409). Landmark-proxy analyses supported these findings (three-month: eight vs. 13 months; p=0.0275; six-month: six vs. 18 months; p=0.0056). Median rates were 0.75 hospitalizations/month, 5.79 hospital days/month, 1.95 RBC units/month, and 0.78 platelet units/month. Patients with the severe composite had higher time-normalized burden, including hospital days/month (7.28 vs. 4.69), RBC units/month (2.48 vs. 1.47), and platelet units/month (2.14 vs. 0.00). Among 57 deaths, complication-related deaths (infection+bleeding) accounted for 52.6%, exceeding progression/acute myeloid leukemia (AML)-attributed deaths (28.1%). In multivariable models, Eastern Cooperative Oncology Group (ECOG) ≥2 and baseline platelets <50×10⁹/L independently predicted the severe composite (OR 9.28 and 4.13, respectively; area under the curve (AUC) 0.759). Dose delay/reduction occurred in 56.8% of HMA-treated patients and was associated with fewer cycles and lower response.

Conclusions

In higher-risk MDS, severe complications are common and define clinically meaningful subgroups with higher care intensity, worse OS, and predominantly complication-related mortality. A composite and count-based framework, paired with patient-month normalization, offers a practical approach to quantify real-world burden and identify patients at the highest risk using readily available baseline variables.

## Introduction

Myelodysplastic neoplasms (MDS) are clonal myeloid disorders characterized by ineffective hematopoiesis, bone marrow dysplasia, peripheral cytopenias, and an inherent risk of transformation to acute myeloid leukemia (AML) [1,2]. In clinical practice, this biological heterogeneity leads to a wide range of disease trajectories. Some patients have an indolent course, with slow progression, lower disease burden, and prolonged survival. Others follow a more aggressive course, with rapid clinical deterioration, early AML transformation, and poor outcomes that often require more intensive therapy. In real-world cohorts, outcomes range from stable disease with near-normal life expectancy and unrelated death (≈40%) to AML transformation (≈25%) or death due to complications of cytopenias and bone marrow failure (≈30%) [3].

Given this variability, risk stratification plays a central role in MDS management [4]. The Revised International Prognostic Scoring System (IPSS-R) is widely used in routine practice and clinical research, providing a structured framework to separate lower-risk from higher-risk disease [5]. Patients classified as high or very high risk represent a particularly vulnerable subgroup. They often present with more profound cytopenias, higher transfusion burden, faster disease progression, and an increased risk of death compared with lower-risk patients. In contemporary reviews, patients with IPSS-R high and very high risk disease have expected median survivals of approximately 1.6 years and 0.8 years, respectively, underscoring the vulnerability of this subgroup [6].

In higher-risk MDS, the disease course is often discussed primarily in terms of two major outcomes: overall survival (OS) and transformation to AML. While these endpoints are important, they do not fully capture the day-to-day clinical burden experienced by patients and physicians. In many cases, the clinical course is dominated not only by progression itself, but also by complications related to persistent cytopenias and treatment exposure. Severe anemia, neutropenia, thrombocytopenia, and immune dysfunction contribute to recurrent infections and bleeding events [7]. These complications often determine treatment suitability, quality of life, and immediate prognosis, even before leukemic transformation occurs.

Among the most clinically relevant complications in higher-risk MDS are infectious and hemorrhagic events. Infections often develop in the setting of neutropenia and neutrophil dysfunction. They can range from relatively mild episodes treated with oral antibiotics to severe infections, sepsis, and even critical illness requiring ICU admission. In real-world data, infections are not only frequent but also clinically important: in a large registry report, infections were the leading cause of death (32%), and pneumonia represented 27.5% of infectious episodes, while sepsis occurred in 8.0% [8]. In the European MDS (EUMDS) registry, infections accounted for 24.6% of deaths in the first year from diagnosis [9]. In addition, baseline neutropenia (absolute neutrophil count (ANC) <0.8 x 10⁹/L) was an independent predictor of infection risk in multivariable analysis.

Hemorrhagic complications, in turn, are driven largely by thrombocytopenia and platelet dysfunction and may vary from minor mucocutaneous bleeding to major bleeding events with life-threatening consequences. Real-world data highlight the clinical impact of bleeding in MDS: in a retrospective hospital network cohort, 11.2% of patients experienced bleeding requiring hospitalization, and bleeding-related deaths occurred even at platelet counts as high as 153 × 10⁹/L [10], underscoring that platelet dysfunction may contribute beyond thrombocytopenia alone. In a large Surveillance, Epidemiology, and End Results (SEER)-Medicare analysis, 1,905 bleeding events occurred among 13,995 MDS patients, and bleeding was strongly associated with subsequent mortality (HR 4.25 for death within three months after the event) [11].

In addition, many patients develop a substantial transfusion burden, requiring repeated RBC and platelet transfusions, which increases hospital visits, supportive care intensity, and impacts overall quality of life [12]. Although these complications are widely recognized in routine hematology practice, they are often described only briefly in studies focused mainly on response rates, progression, or survival.

Furthermore, treatment of higher-risk MDS further complicates the picture. Hypomethylating agents (HMAs) are widely used in patients who are not immediate candidates for intensive chemotherapy or allogeneic transplantation and remain a cornerstone of real-world management. However, the ability to deliver and sustain treatment is shaped by more than treatment itself. Baseline performance status, the depth of cytopenias, infectious and bleeding complications, and the need for hospitalizations or transfusional support all influence whether therapy can be continued as planned.

Real-world studies consistently show that HMA delivery is frequently disrupted. In a population-based study of higher-risk MDS treated with azacitidine, dose reductions occurred in 52.4% of patients, with early discontinuation often attributed to toxicity/adverse events or disease progression [13]. Other real-world series report similar patterns, with schedule modifications in approximately 45% of patients, including dose reductions (≈29%) and treatment delays (≈9%) during azacitidine therapy [14]. Overall, dose delays, dose reductions, and early discontinuation are common in practice and may reflect the interaction between disease biology and complication burden rather than treatment toxicity alone. For this reason, evaluating complications only as isolated events may underestimate their impact on treatment and clinical outcomes.

A complication-oriented approach may therefore provide a more clinically meaningful view of disease burden in higher-risk MDS. Complications should not be considered only as isolated events, such as severe infection, major bleeding, or ICU admission. They should also be viewed as part of an overall burden framework. Composite endpoints, severity-domain counts, and phenotype patterns may better reflect the cumulative strain imposed by the disease.

This perspective is particularly important in real-world cohorts. Patients are often older, have comorbidities, and may not meet the strict selection criteria of clinical trials. In this setting, traditional endpoints such as OS and AML transformation do not fully describe the everyday clinical burden. Measures of healthcare resource use, such as hospitalizations, cumulative hospital days, and transfusion requirements, can add important information. Expressing these variables as rates per patient-month may offer a more accurate representation of burden by accounting for differences in follow-up duration and survival time. Similarly, evaluating complication-related mortality (for example, infection- and bleeding-related deaths) alongside progression-related mortality can clarify our understanding of what most directly drives adverse outcomes in everyday practice.

The main objective of this study was to characterize and quantify the clinical burden of complications in patients with IPSS-R high/very high-risk MDS treated in a tertiary center. Specifically, we assessed infectious, hemorrhagic, and critical-care complications, as well as hospitalization burden, transfusion burden, and cause-of-death patterns. We also developed a complications-centered burden framework based on a severe complication composite and a severe complication count, and examined how these measures related to survival and treatment feasibility, including within the HMA-treated subgroup.

A secondary objective was to explore baseline predictors of severe complication burden using variables readily available in routine practice, such as performance status and blood counts. We hypothesized that a complications-centered framework would identify clinically meaningful subgroups with distinct resource use and treatment feasibility and that accumulating severe complications would be associated with worse outcomes beyond standard risk stratification alone. By focusing on complications as a central feature of disease burden in higher-risk MDS, this study aims to provide a more practice-oriented perspective that may complement traditional prognostic and treatment-response analyses.

## Materials and methods

This was a retrospective, single-center cohort study including adult patients diagnosed with higher-risk MDS and managed at a tertiary center, Coltea Clinical Hospital in Bucharest. Patients were eligible if they had a confirmed diagnosis of MDS, established by bone marrow biopsy and cytogenetic evaluation. Only patients classified as high or very high risk according to the IPSS-R were included. Patients were required to have sufficient clinical follow-up data to allow assessment of complications and outcomes. Cases with incomplete records, preventing complication assessment, were excluded. Institutional Review Board (or Ethics Committee) of Coltea Clinical Hospital issued approval no. 19, dated March 02, 2021.

Morphologic subtypes reported were based on the original diagnostic pathology reports and therefore reflect the WHO 2016 classification, which was the terminology used by the laboratory assessing the bone marrow biopsies at the time of diagnosis. We did not retrospectively remap these categories to the WHO 2022 or International Consensus Classification (ICC) 2022 nomenclature because molecular data were not uniformly available in our cohort. Since the newer classifications place substantial emphasis on molecularly defined MDS entities, retrospective reclassification without complete molecular information could have introduced inaccurate subtype assignment. Therefore, the original WHO 2016 morphologic categories were retained for descriptive purposes, while the manuscript uses the contemporary umbrella term “myelodysplastic neoplasms."

Baseline variables included age, sex, Eastern Cooperative Oncology Group (ECOG) performance status, IPSS-R risk category, peripheral blood counts, and treatment strategy (HMA therapy vs. supportive care). In the HMA-treated subgroup, treatment-related variables included the number of treatment cycles, dose delay/reduction, and best recorded response.

Clinical complication variables included infectious complications (any infection, severe infection, or sepsis); hemorrhagic complications (any bleeding or major bleeding); ICU admission; hospitalizations; cumulative hospital days; and transfusion requirements (RBC and platelet units). Cause of death was categorized as infection, bleeding, progression/AML, or other/unknown.

For reproducibility, key clinical variables were defined as follows. The severe infectious domain included severe infection and/or sepsis. Major bleeding was defined as clinically significant bleeding requiring hospital-based evaluation and/or therapeutic intervention, including transfusional support, invasive hemostatic management, or critical-site bleeding. Dose delay/reduction was defined as any documented postponement of a planned azacitidine cycle or reduction from the intended treatment schedule during follow-up. The best recorded response in the HMA-treated subgroup was abstracted from the medical record as documented by the treating hematologist.

To provide a clinically meaningful complications-centered framework, we selected three severe complication domains that reflected major clinical deterioration in higher-risk MDS and were consistently ascertainable in this retrospective cohort: severe infectious complications, major hemorrhagic complications, and ICU admission as a marker of critical-care-level deterioration. The severe complication composite was defined as the occurrence of at least one of these domains during follow-up. The infectious-severe domain was coded as severe infection and/or sepsis; sepsis was considered nested within the infectious-severe domain and was not counted as a separate additional domain. Accordingly, patients with severe infection alone, sepsis alone, or both were counted once within the infectious-severe domain. A secondary burden metric, severe complication count, was defined as the number of severe domains present (infectious-severe, major bleeding, and ICU), categorized as zero, one, and two-three domains.

For phenotype analyses, patients were additionally grouped into mutually exclusive severe-domain phenotypes: no severe domains, infectious-severe only, hemorrhagic only (major bleeding), and mixed severe (≥2 severe domains). Thus, overlap between infectious-severe, hemorrhagic, and ICU domains was handled analytically through the composite definition, the severe complication count, and the mixed severe phenotype, rather than by counting repeated events within the same domain. A secondary overlap phenotype analysis was also performed using infection/bleeding overlap categories (neither, infection only, bleeding only, both).

The primary focus of the present analysis was the clinical burden and prognostic impact of complications. OS was defined from the study baseline to death from any cause or last follow-up. AML transformation was recorded as a secondary disease outcome but was not the primary focus of the complications-centered analyses. Cause-of-death analyses were performed among deceased patients, and grouped causes of death were also evaluated descriptively as complication-related (infection + bleeding) versus non-complication-related causes. Because infection and bleeding also contribute to the severe complication framework, comparisons between severe complication burden and grouped complication-related death were considered interpretive/descriptive rather than independent causal analyses.

To reduce bias related to differences in follow-up duration, healthcare utilization and transfusion variables were normalized to follow-up time and expressed as rates per patient-month: hospitalizations/month, hospital days/month, RBC units/month, and platelet units/month.

Group comparisons were performed using Mann-Whitney U tests (two groups) or Kruskal-Wallis tests (three or more groups) for continuous variables, and chi-square or Fisher’s exact tests for categorical variables, as appropriate. Survival analyses were performed using the Kaplan-Meier method and compared with the log-rank test. Because complication variables were recorded as cumulative (ever/never during follow-up) rather than time-stamped events, complication-stratified survival analyses were considered exploratory due to potential immortal-time bias. To partially mitigate this, survivor-conditioned landmark-proxy analyses were performed at three months and six months, including only patients alive and under follow-up at the landmark timepoint, with survival time reset from the landmark. All tests were two-sided, and a p-value <0.05 was considered statistically significant. Analyses were performed using Python (Python Software Foundation, Fredericksburg, VA); figures were generated using Matplotlib.

## Results

A total of 72 patients with higher-risk MDS were included in this study. Median age was 71 years (IQR, 65-76), and a slight male predominance was observed (40/72; 55.6%). According to IPSS-R stratification, 33/72 patients (45.8%) were classified as high risk and 39/72 (54.2%) as very high risk. HMA therapy was administered to 44/72 patients (61.1%), while the remaining 28/72 (38.9%) received supportive care only. The median follow-up duration was 11 months. A detailed summary of the epidemiological, morphological, and cytogenetical findings is presented in Table 1.

**Table 1:** Baseline characteristics of the cohort HMA: hypomethylating agents; ECOG: Eastern Cooperative Oncology Group; IPSS-R: Revised International Prognostic Scoring System; WHO: World Health Organization; RAEB II: Refractory Anemia with Excess Blasts type II; RAEB I: Refractory Anemia with Excess Blasts type I; RCMD: Refractory Cytopenia with Multilineage Dysplasia; RARS: Refractory Anemia with Ringed Sideroblasts; ANC: absolute neutrophil count; MDS: myelodysplastic neoplasms; ICC: International Consensus Classification Morphologic categories were retained as originally reported according to the WHO 2016 classification. Formal remapping to WHO 2022/ICC 2022 was not performed because molecular data were not uniformly available in this retrospective cohort, and newer classifications include molecularly defined MDS entities. P-values reflect exploratory comparisons between treatment groups (HMA vs. supportive care): continuous variables were compared using the Mann-Whitney U test, binary variables using Fisher’s exact test, and multi-level categorical distributions using the Pearson χ² test (two-sided).

Characteristic	Overall (N=72)	HMA (n=44)	Supportive (n=28)	p-value
Demographics				
Age, years	71 (65-76)	69.5 (65-74)	73 (66.8-81)	0.0892
Male sex	40 (55.6%)	25 (56.8%)	15 (53.6%)	0.8122
Female sex	32 (44.4%)	19 (43.2%)	13 (46.4%)	0.7985
ECOG ≥2	58 (80.6%)	34 (77.3%)	24 (85.7%)	0.5434
Disease characteristics				
IPSS-R High	33 (45.8%)	16 (36.4%)	17 (60.7%)	0.0624
IPSS-R Very High	39 (54.2%)	28 (63.6%)	11 (39.3%)	0.0545
WHO 2016 classification				0.0562
RAEB II	38 (52.8%)	27 (61.4%)	11 (39.3%)	
RAEB I	22 (30.6%)	9 (20.5%)	13 (46.4%)	
RCMD	11 (15.3%)	8 (18.2%)	3 (10.7%)	
RARS	1 (1.4%)	0 (0.0%)	1 (3.6%)	
Cytogenetic risk				0.1626
Very good	4 (5.6%)	3 (6.8%)	1 (3.6%)	
Good	21 (29.2%)	11 (25.0%)	10 (35.7%)	
Intermediate	29 (40.3%)	22 (50.0%)	7 (25.0%)	
Poor	11 (15.3%)	4 (9.1%)	7 (25.0%)	
Very poor	7 (9.7%)	4 (9.1%)	3 (10.7%)	
Baseline laboratory values				
Bone marrow blasts, %	11 (6-16)	11 (6-16)	8.5 (6-14.5)	0.4005
Hemoglobin, g/dL	7.2 (6.5-8.5)	7.2 (6.7-8.3)	6.9 (6.2-9.6)	0.5401
ANC, ×10⁹/L	1.1 (0.7-2.2)	1.1 (0.7-2.3)	1.1 (0.7-2)	0.7203
ANC <0.8 ×10⁹/L	22 (30.6%)	13 (29.5%)	9 (32.1%)	1.0000
Platelets, ×10⁹/L	80.5 (49.5-121)	83.5 (51.5-117)	77 (48-132)	0.9862
Platelets <50 ×10⁹/L	18 (25.0%)	10 (22.7%)	8 (28.6%)	0.5891
Ferritin at baseline, ng/mL	570 (220-916)	515 (195-914)	680 (278-1042)	0.4861
Follow-up				
Follow-up (evolution), months	11 (6-17)	12 (8.5-23.2)	8.5 (6-12)	0.0251

Complications were common across the cohort. Any infection occurred in 42/72 (58.3%) patients, while severe infection occurred in 26/72 (36.1%) and sepsis in 18/72 (25.0%). Any bleeding occurred in 40/72 (55.6%), and major bleeding in 19/72 (26.4%). ICU admission was documented in 12/72 (16.7%) patients (Table 2).

**Table 2:** Complication profile and absolute clinical burden HMA: hypomethylating agents; ICU: intensive care unit; RBC: red blood cells P-values reflect exploratory comparisons between HMA and supportive care groups: categorical variables were compared using Fisher’s exact test and continuous variables using the Mann-Whitney U test (two-sided).

Characteristic	Overall (N=72)	HMA (n=44)	Supportive (n=28)	p-value
Infectious complications				
Any infection	42 (58.3%)	25 (56.8%)	17 (60.7%)	0.8094
Severe infection	26 (36.1%)	14 (31.8%)	12 (42.9%)	0.4511
Sepsis	18 (25.0%)	10 (22.7%)	8 (28.6%)	0.5891
Hemorrhagic complications				
Any bleeding	40 (55.6%)	27 (61.4%)	13 (46.4%)	0.2341
Major bleeding	19 (26.4%)	13 (29.5%)	6 (21.4%)	0.5855
Critical care				
ICU admission	12 (16.7%)	6 (13.6%)	6 (21.4%)	0.5187
Healthcare utilization and transfusion burden				
Hospitalizations, count	8 (4-11)	9.5 (6.5-12.5)	5 (3-7.2)	0.0024
Hospital days, total	59 (36.8-105)	73.5 (50.8-133)	39.5 (19.8-64)	0.0008
RBC units, total	21.5 (11-37)	25.5 (11.8-37.5)	19 (11-28.5)	0.2099
Platelet units, total	10.5 (0-29)	9 (0-37.2)	10.5 (0-17)	0.5500
Any RBC transfusion (>0 units)	69 (95.8%)	42 (95.5%)	27 (96.4%)	1.0000
Any platelet transfusion (>0 units)	46 (63.9%)	28 (63.6%)	18 (64.3%)	1.0000

The cohort also showed a substantial healthcare resource burden. The median number of overall hospitalizations was 8 (IQR, 4-11), with a median cumulative hospital stay of 59 days (IQR, 36.8-105.2). Median cumulative transfusion burden was 21.5 RBC units (IQR 11-37) and 10.5 platelet units (IQR 0-29). RBC transfusions were nearly universal (69/72, 95.8%), while 46/72 (63.9%) patients received at least one platelet transfusion.

Exploratory Kaplan-Meier analyses were performed to examine OS according to complication categories. OS did not differ significantly across infection severity groups (no infection/non-severe infection/severe infection; log-rank p=0.4198), with median OS of 12, 14, and 10 months, respectively. Similarly, the comparison between patients with no infection and those with severe infection was not statistically significant (median OS 12 vs. 10 months, log-rank p=0.2911).

By contrast, bleeding severity showed a borderline trend toward worse OS (no bleeding/minor bleeding/major bleeding: log-rank p=0.0710), with median OS of 15, 12, and 11 months, respectively. The comparison between minor and major bleeding did not reach statistical significance (median OS 12 vs. 11 months, log-rank p=0.1008).

**Figure 1:**
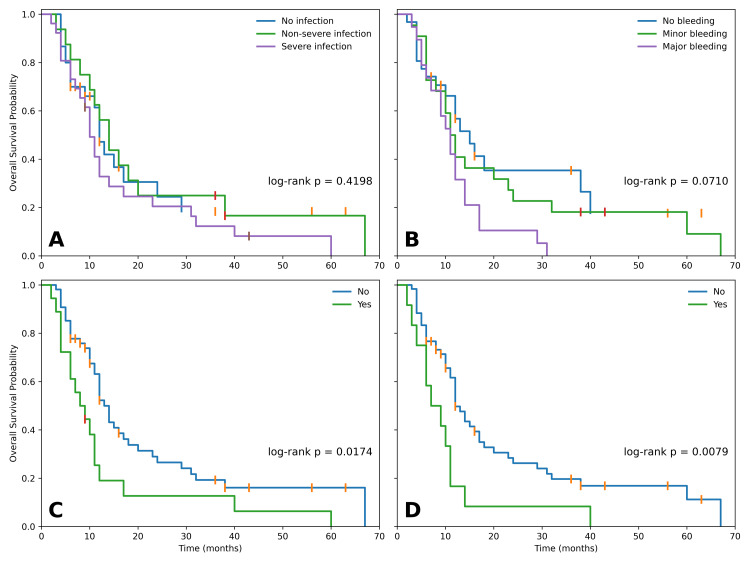
Kaplan-Meier analyses Overall survival stratified by (A) infection severity, (B) bleeding severity, (C) sepsis, (D) ICU admission; log-rank p-values shown.

More severe complication markers showed stronger associations with OS. Patients with sepsis had significantly shorter OS than those without sepsis (median OS 9 vs. 13 months, log-rank p=0.0174). Similarly, ICU admission was associated with inferior OS (median OS 9 vs. 12 months, log-rank p=0.0079) (Figure 1).

Survivor-conditioned landmark-proxy analyses were performed at three months and six months. At the three-month landmark, infection severity remained non-significant (three-group log-rank p=0.5070; no infection vs. severe infection p=0.4646). Bleeding severity showed a persistent trend (three-group p=0.0710; minor vs. major bleeding p=0.0954). In contrast, sepsis remained significantly associated with worse post-landmark OS (median 6 vs. 11 months, log-rank p=0.0461), as did ICU admission (median 7 vs. 10 months, log-rank p=0.0397).

At the six-month landmark, bleeding severity became more clearly associated with subsequent OS (three-group log-rank p=0.0131), and major bleeding was associated with worse OS than minor bleeding (median 6 vs. 8 months, log-rank p=0.0497). ICU admission also remained significantly associated with inferior post-landmark OS (median 5 vs. 11 months, log-rank p=0.0226). Sepsis showed a non-significant adverse trend (log-rank p=0.0687), and infection severity remained non-significant (Table 3).

**Table 3:** Survivor-conditioned landmark-proxy overall survival (OS) analyses Survivor-conditioned landmark-proxy analyses were performed at three and six months. Time zero was reset to the landmark time, and overall survival was estimated using Kaplan-Meier methods. P-values were calculated using two-sided log-rank tests. Median OS values are reported in months and are measured from the landmark.

Landmark	Eligible (n)	Exposure	Overall comparison	Overall p	Pairwise comparison	Pairwise p	Median OS from landmark (months)
3-month	69	Infection severity	3-group	0.5070	No infection vs. severe infection	0.4646	No infection: 9; Severe infection: 8
3-month	69	Bleeding severity	3-group	0.0710	Minor vs. major bleeding	0.0954	No bleeding: 12; Minor: 9; Major: 8
3-month	69	Sepsis	2-group (Yes vs. No)	0.0461	-	-	No sepsis: 11; Sepsis: 6
3-month	69	ICU admission	2-group (Yes vs. No)	0.0397	-	-	No ICU: 10; ICU: 7
6-month	52	Infection severity	3-group	0.3252	No infection vs. severe infection	0.1530	-
6-month	52	Bleeding severity	3-group	0.0131	Minor vs. major bleeding	0.0497	No bleeding: 12; Minor: 8; Major: 6
6-month	52	Sepsis	2-group (Yes vs. No)	0.0687	-	-	-
6-month	52	ICU admission	2-group (Yes vs. No)	0.0226	-	-	No ICU: 11; ICU: 5

The severe complication composite occurred in 38/72 (52.8%) patients. The distribution of severe complication count was as follows: zero domains in 34/72 (47.2%), one domain in 24/72 (33.3%), and two-three domains in 14/72 (19.4%) (Table 4).

**Table 4:** Severe complication burden framework HMA: hypomethylating agents; ICU: intensive care unit P-values reflect exploratory comparisons between HMA and supportive care groups: binary variables were compared using Fisher’s exact test, and severe-count category distributions were compared using the Pearson χ² test (two-sided). *Sepsis was nested within the severe infectious domain and was not counted as a separate domain in the severe complication count. Patients with severe infection alone, sepsis alone, or both contributed to one infectious-severe domain.

Characteristic	Overall (N=72)	HMA (n=44)	Supportive (n=28)	p-value
Severe complication burden framework				
Severe complication composite (Yes)	38 (52.8%)	24 (54.5%)	14 (50.0%)	0.8100
Severe complication domain				
Severe infectious domain* (severe infection and/or sepsis)	26 (36.1%)	14 (31.8%)	12 (42.9%)	0.4511
Major bleeding	19 (26.4%)	13 (29.5%)	6 (21.4%)	0.5855
ICU admission	12 (16.7%)	6 (13.6%)	6 (21.4%)	0.5187
Severe complication count (number of severe domains)				
Severe count category				0.4155
0	34 (47.2%)	20 (45.5%)	14 (50.0%)	
1	24 (33.3%)	17 (38.6%)	7 (25.0%)	
2-3	14 (19.4%)	7 (15.9%)	7 (25.0%)	

**Figure 2:**
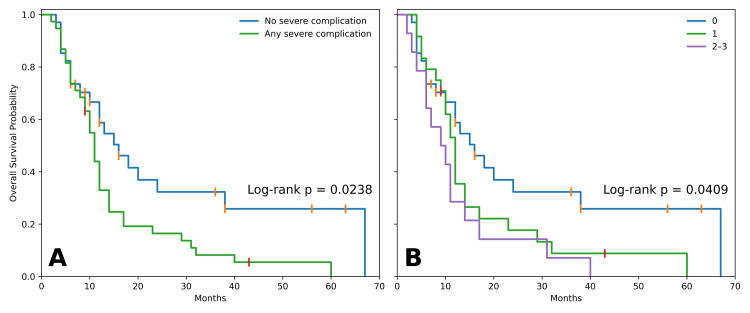
Kaplan-Meier analyses Overall survival stratified by (A) severe complication composite (yes/no) and (B) severe complication count (zero, one, two-three); log-rank p-values shown.

In exploratory standard Kaplan-Meier analyses, patients with the severe complication composite had significantly shorter OS than those without the composite (median OS 11 vs. 16 months, log-rank p=0.0238). Severe complication count also showed an association with OS (median OS 16 months for count zero, 12 months for count one, and nine months for count two-three; log-rank p=0.0409) (Figure 2).

**Figure 3:**
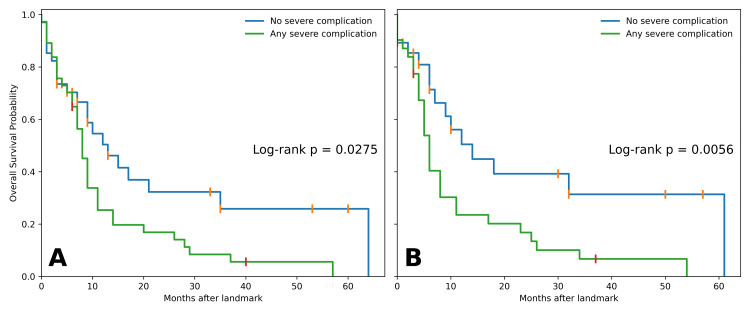
Kaplan-Meier landmark-proxy analyses Overall survival at (A) three-month and (B) six-month landmarks, stratified by severe complication composite; log-rank p-values shown.

These findings were reinforced in landmark-proxy analyses. At the three-month landmark, median OS was eight months in patients with the severe composite versus 13 months in those without (log-rank p=0.0275). At the six-month landmark, the separation was more pronounced (median 6 vs. 18 months, log-rank p=0.0056) (Figure 3).

Across the cohort (total 1,144 patient-months), crude rates per 100 patient-months were 63.6 hospitalizations, 495.4 hospital days, 176.7 RBC units, and 158.5 platelet units. At the patient level, median rates were 0.75 hospitalizations/month (IQR 0.50-0.91), 5.79 hospital days/month (IQR 3.47-8.00), 1.95 RBC units/month (IQR 1.11-3.02), and 0.78 platelet units/month (IQR 0.00-3.16) (Table 5).

**Table 5:** Time-normalized burden (rates per patient-month) Rates are presented as median (IQR). P-values for severe composite comparisons were calculated using the Mann-Whitney U test, and p-values across severe-count categories were calculated using the Kruskal-Wallis test (two-sided).

Metric (per patient-month)	Overall (N=72)	Severe composite: No	Severe composite: Yes	p (composite)	Severe count: 0	Severe count: 1	Severe count: 2-3	p (count)
Hospitalizations/month	0.75 (0.50-0.91)	0.69 (0.50-0.84)	0.79 (0.62-0.92)	0.0695	0.69 (0.50-0.84)	0.77 (0.50-0.91)	0.85 (0.73-1.00)	0.0819
Hospital days/month	5.79 (3.47-8.00)	4.69 (2.53-6.69)	7.28 (5.57-8.89)	0.0002	4.69 (2.53-6.69)	6.46 (5.48-8.50)	8.42 (6.15-10.57)	0.0004
RBC units/month	1.95 (1.11-3.02)	1.47 (0.68-2.92)	2.48 (1.44-3.99)	0.0190	1.47 (0.68-2.92)	2.36 (1.40-2.99)	2.69 (1.80-4.79)	0.0342
Platelet units/month	0.78 (0.00-3.16)	0.00 (0.00-0.81)	2.14 (0.72-5.56)	0.0001	0.00 (0.00-0.81)	1.39 (0.49-4.50)	3.02 (1.48-5.56)	0.0004

Compared with patients without the severe complication composite, those with the severe composite had a significantly higher time-normalized care burden, including more hospital days/month (7.28 vs. 4.69, p=0.00017), higher RBC transfusion intensity (2.48 vs. 1.47 units/month, p=0.0190), and higher platelet transfusion intensity (2.14 vs. 0.00 units/month, p=0.00013). Hospitalizations/month were also higher (0.79 vs. 0.69), but the difference was not statistically significant (p=0.0695).

A similar pattern was observed across severe complication count categories (zero vs. one vs. two-three), with increasing hospital days/month (4.69 vs. 6.46 vs. 8.42; p=0.00040), RBC units/month (1.47 vs. 2.36 vs. 2.69; p=0.0342), and platelet units/month (0.00 vs. 1.39 vs. 3.02; p=0.00039) (Table 5).

To further characterize complication patterns, mutually exclusive severe-domain phenotypes were defined as: no severe domains, infectious-severe only, hemorrhagic (major bleeding) only, and mixed severe (≥2 severe domains). The phenotype distribution was 34/72 (47.2%), 14/72 (19.4%), 10/72 (13.9%), and 14/72 (19.4%), respectively (Table 6).

**Table 6:** Severe-domain phenotype distribution and time-normalized burden Rates are presented as median (IQR). Overall comparisons across phenotype groups were performed using the Kruskal-Wallis test (two-sided).

Phenotype distribution	n (%)	Hospital days/month	RBC units/month	Platelet units/month	p-value
No severe domains	34 (47.2%)	4.69 (2.53-6.69)	1.47 (0.68-2.92)	0.00 (0.00-0.81)	-
Infectious-severe only	14 (19.4%)	6.76 (5.63-8.50)	2.51 (1.99-3.33)	0.94 (0.17-2.51)	
Hemorrhagic only (major bleeding)	10 (13.9%)	6.11 (5.33-7.34)	1.39 (0.92-2.27)	3.96 (0.68-5.92)	
Mixed severe (≥2 domains)	14 (19.4%)	8.42 (6.15-10.57)	2.69 (1.80-4.79)	3.02 (1.48-5.56)	
Overall comparison (Kruskal-Wallis)	-	-	-	-	Hospital days/month 0.0013; RBC/month 0.0237; Platelets/month 0.0007

Exploratory OS analysis across severe-domain phenotypes showed a non-significant trend (log-rank p=0.0635), with median OS of 16 months (no severe domains), 12 months (infectious-severe only), 12 months (hemorrhagic only), and nine months (mixed severe), suggesting the poorest survival in patients with mixed severe phenotypes.

Phenotype-specific analyses of time-normalized care burden showed significant differences, including hospital days/month (p=0.0013), RBC units/month (p=0.0237), and platelet units/month (p=0.00068). Overall, patients with mixed severe phenotypes had the highest burden across domains. Infectious-severe phenotypes were associated with greater hospital-day burden and RBC transfusion intensity, whereas hemorrhagic phenotypes were associated with the highest platelet transfusion intensity (Table 6).

Among the 57 deaths, the recorded causes of death were bleeding in 17 (29.8%), progression/AML in 16 (28.1%), infection in 13 (22.8%), and other/unknown in 11 (19.3%). Most cases in the other/unknown category (9/11) were cardiovascular in nature, including events related to advanced age and cardiac decompensation (often in the context of severe anemia). When grouped, complication-related deaths (infection + bleeding) accounted for 30/57 (52.6%), exceeding progression/AML-attributed deaths (16/57, 28.1%) (Table 7).

**Table 7:** Cause-of-death distribution AML: acute myeloid leukemia Cause of death is reported among deaths (n=57). Percentages are column percentages among deaths. Grouped cause of death classifies infection and bleeding as complication-related mortality. In the Other/Unknown category, 9/11 deaths were described as cardiovascular in nature.

Variable	n	%
Cohort context		
Total cohort size	72	100
Deaths	57	79.2
Alive at last follow-up	15	20.8
Cause-of-death categories		
Bleeding	17	29.8
Progression/AML	16	28.1
Infection	13	22.8
Other/Unknown	11	19.3
Grouped cause of death		
Complication-related (infection + bleeding)	30	52.6
Progression/AML	16	28.1
Other/Unknown	11	19.3

The grouped cause-of-death distribution differed significantly by severe composite status (2×3 comparison p<0.0001), as did the four-category cause-of-death distribution (2×4 p<0.0001). In a Fisher's exact comparison (complication-related vs. non-complication-related death), the severe composite was strongly associated with complication-related death attribution (OR 82.86, p<0.0001) (Table 8). However, because the severe complication composite includes infection- and bleeding-related domains and the grouped mortality endpoint also includes infection and bleeding, these comparisons should be interpreted cautiously as descriptive concordance rather than as an independent association. They are presented to illustrate the alignment between severe complication burden and complication-attributed death patterns within the cohort.

**Table 8:** Association with severe complication composite (among deaths) COD: cause of death; AML: acute myeloid leukemia P-values for distributional comparisons by severe composite status were calculated using Pearson χ² tests; the binary comparison (complication-related vs. non-complication-related death) used Fisher’s exact test.

Comparison	Test	Result
Grouped COD distribution (complication-related vs. progression/AML vs. other/unknown)	Pearson χ² (2×3)	p<0.0001
4-category COD distribution (bleeding/infection/progression-AML/other-unknown)	Pearson χ² (2×4)	p<0.0001
Complication-related vs. non-complication-related death	Fisher’s exact	OR 82.86; p<0.0001

All 44 HMA-treated patients received azacitidine. Dose delay or dose reduction occurred in 25/44 (56.8%) patients. Median number of administered cycles was 5.5 (IQR 2.0-12.0), with 26/44 (59.1%) receiving at least four cycles and 22/44 (50.0%) receiving at least six cycles (Table 9).

**Table 9:** HMA subgroup characteristics HMA: hypomethylating agents; IQR: interquartile range; CR: complete remission; PR: partial remission; SD: stable disease; PD: progressive disease Values are n/N (%) or median (IQR).

Characteristic	HMA subgroup (n=44)
Dose delay/reduction	25 (56.8%)
Cycles administered, median (IQR)	5.5 (2-12)
≥4 cycles	26 (59.1%)
≥6 cycles	22 (50.0%)
Best response: CR	3 (6.8%)
Best response: PR	6 (13.6%)
Best response: SD	27 (61.4%)
Best response: PD	8 (18.2%)
Overall response rate (CR/PR)	9 (20.5%)
Disease control rate (CR/PR/SD)	36 (81.8%)

Patients with dose delay/reduction received fewer cycles than those without delay/reduction (median 3.0 (IQR 1.0-6.0) vs. 8.0 (IQR 5.5-15.0), p=0.012). They were also less likely to receive ≥4 cycles (40.0% vs. 84.2%, p=0.0050) or ≥6 cycles (32.0% vs. 73.7%, p=0.0139). The overall response rate was lower in the delay/reduction group (8.0% vs. 36.8%, Fisher's p=0.0270) (Table 10).

**Table 10:** Treatment feasibility and burden according to dose delay/reduction in the HMA subgroup IQR: interquartile range; ORR: overall response rate; CR: complete remission; PR: partial remission; SD: stable disease; PD: progressive disease; HMA: hypomethylating agents Values are n/N (%) or median (IQR). For two-group comparisons, p-values were calculated using Fisher’s exact test for categorical variables and the Mann-Whitney U test for continuous variables.

Characteristic	Delay/reduction (Yes)	Delay/reduction (No)	p-value
Group size	n=25	n=19	-
Cycles administered, median (IQR)	3 (1-6)	8 (5.5-15)	0.0121
≥4 cycles	10 (40.0%)	16 (84.2%)	0.0050
≥6 cycles	8 (32.0%)	14 (73.7%)	0.0139
ORR (CR/PR)	2 (8.0%)	7 (36.8%)	0.0270
4-category response distribution (CR/PR/SD/PD)	-	-	0.0789
Severe infection	10 (40.0%)	4 (21.1%)	0.2109
Sepsis	8 (32.0%)	2 (10.5%)	0.1483
Major bleeding	9 (36.0%)	4 (21.1%)	0.3346
ICU admission	5 (20.0%)	1 (5.3%)	0.2131
Severe composite	16 (64.0%)	8 (42.1%)	0.2227
Hospital days/month	6.17 (5.54-7.89)	6.67 (4.15-8)	0.7944
RBC units/month	2.55 (1.44-3)	1.42 (0.7-2.71)	0.2134
Platelet units/month	0.7 (0-3.67)	0.71 (0.08-3.3)	0.6186
Hospitalizations/month	0.78 (0.6-0.91)	0.83 (0.69-0.97)	0.6954

Within the HMA subgroup, patients with the severe complication composite showed a higher frequency of dose delay/reduction (66.7% vs. 45.0%, p=0.223) and lower treatment exposure, reflected by fewer treatment cycles (median 3.5 vs. 6.0, p=0.181), but these differences did not reach statistical significance. In contrast, severe complication burden was more clearly associated with higher concurrent care and transfusion burden even among HMA-treated patients. Patients with the severe complication composite had higher total platelet transfusion requirements (30.0 vs. 1.0 units, p=0.0051), more hospital days per month (7.3 vs. 5.3, p=0.0017), higher RBC transfusion intensity (2.6 vs. 1.3 units/month, p=0.0178), and higher platelet transfusion intensity (2.5 vs. 0.1 units/month, p=0.0029) (Table 11). These HMA subgroup findings should be interpreted as exploratory, given the limited sample size.

**Table 11:** Severe complication composite in the HMA subgroup IQR, interquartile range; ORR: overall response rate; CR: complete remission; PR: partial remission; HMA: hypomethylating agents Values are n/N (%) or median (IQR). For two-group comparisons, p-values were calculated using Fisher’s exact test for categorical variables and the Mann-Whitney U test for continuous variables.

Characteristic	Severe composite (No)	Severe composite (Yes)	p-value
Group size	n=20	n=24	-
Dose delay/reduction, yes	9 (45.0%)	16 (66.7%)	0.2227
Cycles administered, median (IQR)	6 (3-15)	3.5 (2-8)	0.1811
≥4 cycles	14 (70.0%)	12 (50.0%)	0.2268
≥6 cycles	12 (60.0%)	10 (41.7%)	0.3640
ORR (CR/PR)	6 (30.0%)	3 (12.5%)	0.2607
Platelet units total	1 (0-14.5)	30 (4.5-69)	0.0051
Hospital days/month	5.3 (2.8-6.8)	7.3 (6-9.3)	0.0017
RBC units/month	1.3 (0.6-2.5)	2.6 (1.6-4.1)	0.0178
Platelet units/month	0.1 (0-0.7)	2.5 (0.5-5.8)	0.0029

A similar graded pattern was observed across severe complication count categories within the HMA subgroup. The frequency of dose delay/reduction increased from 45.0% in patients with severe count zero, to 58.8% in those with severe count one, and 85.7% in those with severe count two-three, with a near-significant trend (p=0.075). In parallel, significant gradients were observed for hospital days/month, RBC units/month, and platelet units/month (all p < 0.05). However, because the HMA subgroup was small and the severe count two-three subgroup included only seven patients, these findings should be interpreted as hypothesis-generating. They support a possible relationship between accumulating severe complications, greater care burden, and reduced treatment feasibility but do not establish a causal effect (Table 12).

**Table 12:** Treatment feasibility and burden according to severe complication count in the HMA subgroup HMA: hypomethylating agents; IQR: interquartile range; ORR: overall response rate; CR: complete remission; PR: partial remission Values are n/N (%) or median (IQR). For three-group severe count comparisons, categorical variables were compared using Pearson χ² tests and continuous variables using the Kruskal-Wallis test. Trend p-values reflect Spearman's rank correlation using the exact severe complication count (0-3).

Characteristic	Severe count 0	Severe count 1	Severe count 2-3	p (overall)	p (trend)
Group size	n=20	n=17	n=7	-	-
Dose delay/reduction, yes	9 (45.0%)	10 (58.8%)	6 (85.7%)	0.1696	0.0753
Cycles administered, median (IQR)	6 (3-15)	4 (2-11)	3 (1.5-6.5)	0.3812	0.1648
ORR (CR/PR)	6 (30.0%)	2 (11.8%)	1 (14.3%)	0.3547	0.1929
Hospital days/month	5.27 (2.76-6.78)	6.77 (6.08-8.56)	8.33 (6.73-10.49)	0.0061	0.0009
RBC units/month	1.27 (0.61-2.55)	2.42 (1.42-2.83)	2.64 (2.23-4.58)	0.0265	0.0056
Platelet units/month	0.08 (0-0.68)	1.79 (0-5.67)	3.33 (1.39-9)	0.0083	0.0012
Platelet units total	1 (0-14.5)	37 (0-72)	28 (13.5-35)	0.0187	0.0055

An exploratory multivariable logistic regression model was constructed for the severe complication composite (defined as severe infection/sepsis, major bleeding, and/or ICU admission), including age (per 10 years), sex, ECOG performance status (≥2 vs. <2), risk category (very high vs. high), and baseline platelet count (<50 ×10⁹/L vs. ≥50 ×10⁹/L). In this model, ECOG ≥2 and platelet count <50 ×10⁹/L were independently associated with the severe complication composite, with odds ratios of 9.28 (95% CI 1.49-57.95, p=0.017) and 4.13 (95% CI 1.11-15.45, p=0.035), respectively. Age, sex, and risk category were not independently significant predictors. The model showed moderate discriminative ability (AUC 0.759) (Table 13). Given the modest sample size and wide confidence intervals, these findings should be interpreted as hypothesis-generating.

**Table 13:** Exploratory predictors of severe complication burden OR: odds ratio; CI: confidence interval; ECOG: Eastern Cooperative Oncology Group; IPSS-R: Revised International Prognostic Scoring System; N: number; AUC: area under the curve P-values were derived from Wald tests of regression coefficients in multivariable logistic regression (severe complication composite) and proportional-odds ordinal logistic regression (severe complication count categories) (two-sided). Predictor analyses should be interpreted as exploratory because of the modest sample size and wide confidence intervals; external validation is required.

Predictor	Severe composite OR (95% CI)	p-value	Severe count common OR (95% CI)	p-value (ordinal)
Age (per 10 years)	0.70 (0.42-1.19)	0.1924	0.69 (0.42-1.13)	0.1393
Male sex	0.76 (0.27-2.17)	0.6088	1.11 (0.44-2.81)	0.8264
Female sex	1.32 (0.46-3.70)	0.6088	0.90 (0.36-2.27)	0.8264
ECOG ≥2	9.28 (1.49-57.95)	0.0171	10.46 (1.75-62.61)	0.0101
IPSS-R Very High (vs. High)	1.39 (0.49-3.95)	0.5361	1.71 (0.66-4.43)	0.2651
Platelets <50×10⁹/L	4.13 (1.11-15.45)	0.0349	3.19 (1.12-9.11)	0.0300
Model N (complete cases)	72	­­­­-	72	-
Model discrimination (AUC)	0.759	-	-	-

In exploratory component-specific analyses, ECOG ≥2 was associated with the severe infectious phenotype (severe infection and/or sepsis; OR 7.85, 95% CI 1.01-61.11, p=0.049), while platelets <50 ×10⁹/L was strongly associated with major bleeding (OR 8.62, 95% CI 2.40-31.02, p=0.0010).

## Discussion

This study was designed to describe the real-world complications burden in patients with IPSS-R high/very high-risk MDS, and to show why complications deserve to be treated as central clinical endpoints rather than “secondary events.” In our cohort, complications were common and often severe. Over half of the patients developed infection, and more than one-third developed severe infection. Major bleeding and ICU admission were also frequent. These events translated into a high resource burden, with substantial hospitalization time and transfusion requirements. Importantly, when complications were summarized through a severe complication composite and a severe complication count, we identified clinically distinct subgroups. The subgroups differed in survival, time-normalized resource use, and cause-of-death patterns. 

One of the main contributions of this study is the use of a complication burden framework, rather than evaluating each complication in isolation. In clinical practice, patients rarely experience a single severe event. Severe infection, sepsis, major bleeding, and ICU admission often cluster or occur sequentially. A composite endpoint captures this reality and improves interpretability. The count-based metric (zero, one, or two-three severe domains) adds a clear dose-response dimension and makes the concept of burden more clinically intuitive.

In our cohort, both the severe complication composite and the severe complication count were associated with worse OS in exploratory Kaplan-Meier analyses and in survivor-conditioned landmark-proxy analyses. Although cumulative (ever/never) complication coding has limitations, the consistency of findings across standard and landmark-proxy approaches supports the conclusion that severe complications are not incidental. Rather, they identify a subgroup with distinctly poorer subsequent outcomes.

Infections were especially prominent in our cohort (n=42/72; 58.3%). This is consistent with large registry data suggesting that infections remain a major threat in MDS and may represent a leading cause of death in real-world practice. In the Düsseldorf MDS Registry report, infections were the most frequent cause of death [8]. Pneumonia was the dominant infection type, and sepsis represented a smaller but clinically important fraction. Our findings align to some extent: pneumonia was present in 8/42 (19%) cases of documented infection and sepsis in 18/72 (25%) patients. Although direct comparisons across cohorts are limited by differences in case mix, definitions, and treatment exposure, the general message is consistent. Infections are frequent, severe infections are not rare, and infection-related outcomes can meaningfully shape prognosis.

Severe infections were a major component of the severe complication composite. The fact that infection severity groups did not consistently separate survival in standard Kaplan-Meier analyses likely reflects methodological constraints. In our dataset, infections were recorded as cumulative exposures (ever/never) rather than time-stamped events. This creates the risk of immortal-time bias, because patients must survive long enough to be “classified” as having a complication. Our landmark-proxy analyses partially addressed this limitation and suggested that higher infection markers, particularly sepsis, were more consistently associated with worse subsequent outcomes.

These findings are in line with contemporary work showing that infection risk in MDS is influenced by more than a single baseline ANC value. Host immune dysfunction, qualitative neutrophil defects, treatment exposure, and early-cycle vulnerability all contribute. Recent reviews also emphasize that the highest-risk period may occur during severe neutropenia and early treatment cycles, when antibacterial prophylaxis may be appropriate for selected higher-risk patients [15]. This supports a practical interpretation of our data. Infection risk should be approached as dynamic, not static. It may be better captured by severe-event indicators (sepsis, ICU) and by cumulative burden frameworks than by a single baseline value.

Hemorrhagic complications were also frequent in our cohort, including a substantial rate of major bleeding. Importantly, bleeding severity showed a clearer relationship with post-landmark survival in later landmarks (six-month landmark), suggesting that hemorrhagic complications may act as a marker of fragility and late-course marrow failure. This fits with the literature describing that bleeding risk in MDS is not explained by thrombocytopenia alone. Platelet dysfunction is common, and bleeding can occur even at platelet counts that would not typically be considered “catastrophic” in other settings [10]. A recent detailed review of thrombocytopenia in MDS emphasizes both the prevalence of platelet dysfunction and the clinical reality that bleeding may occur even when platelet counts are not extremely low [16].

Our finding that baseline platelets <50 × 10⁹/L predicted major bleeding and severe composite burden is also consistent with real-world thrombocytopenia-focused cohorts. In a study focusing on MDS patients with platelets <50 × 10⁹/L, bleeding signs were reported in roughly one-fifth of thrombocytopenic patients [17]. This supports the clinical intuition behind our predictors model. Severe thrombocytopenia is not just a laboratory abnormality. It is a clinically relevant marker of hemorrhagic vulnerability and overall marrow failure burden.

A major strength of this work is the use of rates per patient-month for hospitalizations, hospital days, and transfusion intensity. Absolute event counts can be misleading. Patients who die early may appear to have a lower burden simply because they had less time to accumulate events. In contrast, time-normalized measures better reflect intensity. They also align more closely with clinical workload and health-system impact. We observed higher hospital days/month and higher RBC and platelet units/month in patients with severe composite complications, supporting a clear association between severe complications and ongoing care intensity.

These findings are consistent with real-world evidence showing that higher-risk MDS often requires substantial supportive care and frequent hospital interaction. In a study assessing RBC transfusion burden, a meaningful proportion of patients required inpatient hospitalization within a short observation window, and emergency transfusions for symptomatic anemia were a common reason for admission [18]. Systematic reviews of the economic and healthcare burden in MDS similarly show that transfusion dependence is associated with increased hospitalizations and higher resource use [19]. Our results extend this by showing that the burden is not only high but also stratified by severe complication burden and remains evident even after time normalization.

The phenotype analysis adds an additional layer. The mixed severe phenotype (≥2 severe domains) showed the highest overall resource burden and the poorest survival, even if the survival comparison was borderline in standard Kaplan-Meier analyses. In contrast, the infectious-severe and hemorrhagic phenotypes showed different burden profiles. The infectious-severe phenotype was associated with more hospital days and higher RBC transfusion intensity, whereas the hemorrhagic phenotype was characterized by the highest platelet transfusion intensity.

This is clinically helpful because it reflects patterns physicians recognize at the bedside. It also suggests that different patients may follow distinct “complication pathways," which could guide surveillance and supportive care strategies. Infection-dominant phenotypes might benefit most from early broad-spectrum therapy protocols and selective prophylaxis in high-risk periods. Hemorrhagic phenotypes may require closer platelet monitoring, individualized transfusion thresholds, and attention to qualitative platelet dysfunction.

In our cohort, complication-related deaths (infection + bleeding) exceeded progression/AML-attributed deaths. This is consistent with registry observations that infection and other marrow-failure complications contribute substantially to mortality in MDS, sometimes exceeding AML transformation as a direct cause of death. A recent analysis of low-risk progression patterns reported that a substantial share of deaths occurred from cytopenia-related complications rather than progression, reinforcing that complications can be direct causes of death across the MDS spectrum [20]. Although our cohort is higher-risk and has different competing risks, the clinical message is similar. For many patients, the immediate threat is not only transformation. It is infection, hemorrhage, and end-organ decompensation on a background of cytopenias.

We acknowledge an inherent overlap in definitions because the severe complication composite includes infection- and bleeding-related domains, and the grouped cause-of-death endpoint also includes infection and bleeding. Therefore, the observed relationship between severe complication burden and complication-related death should be interpreted cautiously and descriptively, as evidence of internal concordance rather than as an independent or causal association. In this study, the cause-of-death analysis was intended to contextualize the clinical relevance of the burden framework, not to establish predictive specificity for death attribution.

We also observed a notable cardiovascular contribution within the “other/unknown” category. This is not surprising in an older population with severe anemia and repeated physiologic stress. Contemporary population data continue to explore the link between MDS and cardiovascular events, suggesting that cardiovascular risk is a meaningful competing cause of morbidity and mortality in MDS [21]. This supports a balanced interpretation of our cause-of-death findings. In higher-risk MDS, death mechanisms are often mixed. Complications and comorbid biology may coexist and reinforce each other.

Our HMA subgroup findings suggest that treatment feasibility may be related to complication burden, but these observations should be interpreted cautiously, given the limited subgroup size. In our cohort, dose delays and dose reductions were common and overall were associated with fewer administered cycles and a lower complete remission/partial remission (CR/PR) rate. This aligns with real-world HMA data showing that many patients do not receive prolonged, uninterrupted therapy. In a real-world analysis of azacitidine use, fewer cycles and suboptimal persistence were associated with worse outcomes, suggesting that treatment feasibility is not only a process metric but may also influence survival [22]. However, within our own HMA subgroup, the comparisons linking severe complication burden with dose delay/reduction and lower treatment exposure did not consistently reach statistical significance and should therefore be viewed as exploratory rather than confirmatory.

Real-world effectiveness cohorts also show that outcomes remain poor overall, despite azacitidine being a standard option in higher-risk MDS. Large chart-review studies highlight the gap between “trial intent” and “real-world delivery" and reinforce the need to understand why patients discontinue or de-intensify therapy [23]. Our results add a practical interpretation to this gap, but only at a hypothesis-generating level. In our cohort, a greater severe complication burden co-occurred with higher healthcare utilization and showed directional trends toward more dose delay/reduction and lower treatment exposure. These findings are compatible with the hypothesis that complications may contribute to treatment disruption through hospital admissions, infections, bleeding events, and progressive physiologic deconditioning. However, given the small subgroup sizes, this interpretation should not be considered causal, and what appears as “treatment intolerance” may reflect a combination of disease-driven fragility, treatment-related factors, and baseline patient vulnerability.

In exploratory multivariable analysis, ECOG ≥2 and platelets <50 × 10⁹/L emerged as candidate predictors of the severe composite. These findings are clinically plausible and potentially relevant in practice, because both variables are readily available at baseline. However, the confidence intervals were wide, reflecting the limited sample size of this single-center cohort. Accordingly, these results should be interpreted as hypothesis-generating rather than definitive, and they should not be considered a validated prediction model.

The performance status signal also aligns with critical-care literature in hematologic malignancies. Reviews of ICU admission in hematologic cancers highlight that outcomes are worse in patients with poor functional status and higher illness severity, and they support structured, time-limited ICU trials with early reassessment [24]. In our cohort, ICU admission and sepsis were among the strongest markers of poor subsequent survival.

The absence of an independent association between baseline ANC and severe complication burden in our cohort deserves comment. EUMDS data in lower-risk MDS found ANC <0.8 × 10⁹/L to be an independent infection-risk predictor [9]. In our higher-risk cohort, baseline ANC may have been less informative because infection risk is influenced by multiple factors beyond a single baseline value, including neutrophil dysfunction, dynamic cytopenia during treatment cycles, and competing severe domains (major bleeding, ICU). This does not argue against ANC as a clinically relevant marker. Instead, it suggests that for higher-risk cohorts, a broader burden framework may capture vulnerability more effectively than any single baseline cytopenia marker.

This study has several limitations. First, it is retrospective and single-center, which introduces selection bias and limits generalizability. Second, complication variables were recorded as cumulative (ever/never) rather than time-stamped events, which limits causal inference and introduces potential immortal-time bias in complication-oriented survival analyses. We attempted to reduce this with landmark-proxy analyses, but these remain approximations. Third, cause-of-death attribution is imperfect in retrospective datasets and may be influenced by documentation quality. Fourth, the modest sample size limited the complexity and precision of multivariable modeling, resulting in wide confidence intervals around several predictor estimates; therefore, the predictor analyses should be regarded as exploratory. The HMA-treated subgroup was also relatively small, and some treatment-feasibility subgroup comparisons were based on very small cell counts, limiting precision and precluding causal inference. Finally, molecular data were not incorporated into the predictors framework, which may further refine risk stratification in contemporary practice.

Despite these limitations, our study provides a clinically useful framework for summarizing complications in higher-risk MDS. The severe complication composite and severe complication count capture real-world burden in a simple and interpretable way. Rates per patient-month allow a more impartial comparison of burden across patients with different survival times and follow-up durations. In addition, phenotype groupings help translate complex clinical courses into recognizable patterns that can be discussed and compared across cohorts.

Future work should aim to collect event timing and recurrence for major complications. This would enable time-dependent modeling and formal landmark analyses and would strengthen causal interpretation. The exploratory predictor findings reported here require external validation in larger, multicenter datasets before they can be considered for broader risk stratification use. It would also be valuable to integrate molecular risk and inflammatory or immune markers, given the likely interaction between clonal biology, immune dysfunction, and susceptibility to infection and bleeding. Finally, complications-centered endpoints may be useful for evaluating supportive care interventions, including targeted prophylaxis strategies during clearly defined high-risk periods.

## Conclusions

In higher-risk MDS, severe complications are common and clinically decisive. They are associated with higher care intensity, worse survival, and a large proportion of complication-related mortality. A severe complication composite and severe complication count provided a practical way to summarize this burden and identified subgroups with distinctly worse survival and higher time-normalized care burden. In the HMA-treated subgroup, severe complications were associated with greater concurrent care burden and showed exploratory signals of reduced treatment feasibility, but these findings require cautious interpretation. ECOG performance status and severe thrombocytopenia emerged as exploratory baseline predictors of severe complication burden in this cohort. These findings support the inclusion of complication burden measures as meaningful endpoints in higher-risk MDS research and as practical tools for supportive care in everyday practice.
